# Advancements in lead therapeutic phytochemicals polycystic ovary syndrome: A review

**DOI:** 10.3389/fphar.2022.1065243

**Published:** 2023-01-09

**Authors:** Er-Dan Luo, Hai-Mei Jiang, Wei Chen, Yao Wang, Mi Tang, Wen-Mei Guo, Hao-Yang Diao, Ning-Yuan Cai, Xiao Yang, Ying Bian, Sha-Sha Xing

**Affiliations:** ^1^ GCP Institution, Chengdu Women’s and Children’s Central Hospital, School of Medicine, University of Electronic Science and Technology of China, Chengdu, China; ^2^ School of Pharmacy, Chengdu University of Traditional Chinese Medicine, Chengdu, China; ^3^ Traditional Chinese Medicine Department, Chengdu Women’s and Children’s Central Hospital, School of Medicine, University of Electronic Science and Technology of China, Chengdu, China; ^4^ State Key Laboratory of Quality Research in Chinese Medicine, Institute of Chinese Medical Sciences, University of Macau, Chengdu, China

**Keywords:** polycystic ovary syndrome, phytochemicals, flavonoid, polyphenol, alkaloid

## Abstract

Polycystic ovary syndrome (PCOS) is one of the most common endocrine diseases in women of reproductive age and features complex pathological symptoms and mechanisms. Existing medical treatments have, to some extent, alleviated the deterioration of PCOS. However, these strategies only temporarily control symptoms, with a few side effects and no preventive effect. Phytochemicals extracted from medicinal herbs and plants are vital for discovering novel drugs. In recent years, many kinds of research have proven that phytochemicals isolated from traditional Chinese medicine (TCM) and medicinal plants show significant potential in preventing, alleviating, and treating PCOS. Nevertheless, compared to the abundance of experimental literature and minimal specific-topic reviews related to PCOS, there is a lack of systematic reviews to summarize these advancements in this promising field. Under this background, we systematically document the progress of bioactive phytochemicals from TCM and medicinal plants in treating PCOS, including flavonoids, polyphenols, and alkaloids. According to the literature, these valuable phytochemicals demonstrated therapeutic effects on PCOS supported by *in vivo* and *in vitro* experiments, mainly depending on anti-inflammatory, antioxidation, improvement of hormone disorder and insulin resistance (IR), and alleviation of hyperinsulinemia. Based on the current progress, future research directions should emphasize 1) exploring bioactive phytochemicals that potentially mediate bone metabolism for the treatment of PCOS; 2) improving unsatisfactory bioavailability by using advanced drug delivery systems such as nanoparticles and antibody-conjugated drugs, as well as a chemical modification; 3) conducting in-depth research on the pathogenesis of PCOS to potentially impact the gut microbiota and its metabolites in the evolution of PCOS; 4) revealing the pharmacological effects of these bioactive phytochemicals on PCOS at the genetic level; and 5) exploring the hypothetical and unprecedented functions in regulating PCOS by serving as proteolysis-targeting chimeras and molecular glues compared with traditional small molecule drugs. In brief, this review aims to provide detailed mechanisms of these bioactive phytochemicals and hopefully practical and reliable insight into clinical applications concerning PCOS.

## 1 Introduction

Polycystic ovary syndrome (PCOS) is one of the most common endocrine diseases with a heterogeneous genetic condition in women of reproductive age (15–49 years) (Palomba et al., 2015; Balen et al., 2016; Escobar-Morreale, 2018). Data from the Global Burden of Disease (GBD) in 2019 showed that the incidence of PCOS increased by 30.4% between 1990 and 2019, reaching 66 million cases globally in 2019 (Murray et al., 2020). According to the authoritative Rotterdam criteria formulated by the American Society for Reproductive Medicine (ASRM) and European Society of Human Reproduction and Embryology (ESHRE) in 2003 (Rotterdam ESHRE/ASRM-Sponsored PCOS consensus workshop group), PCOS can be diagnosed with two symptoms of the following three: 1) chronic oligo-anovulation or anovulation; 2) clinical or biochemical hyperandrogenism; and 3) polycystic ovarian morphology. With the development of clinical research on POCS, according to recommendations from the International Evidence-based Guideline for the Assessment and Management of Polycystic Ovary Syndrome from 2018 (Teede et al., 2018), PCOS has been further distinguished into four phenotypes: phenotype A manifested as the excess of androgens, ovulatory dysfunction, and polycystic ovary on ultrasound; phenotype B manifested as the excess of androgens and ovulatory dysfunction; phenotype C manifested as the excess of androgens and polycystic ovary on ultrasound; and phenotype D manifested as ovulatory dysfunction and polycystic ovary on ultrasound. Although the guidelines mentioned above permit the diagnosis of PCOS, the clinical manifestations of PCOS are complex and reference multiple symptoms, such as ovarian enlargement (Azziz et al., 2016; Escobar-Morreale, 2018), hyperandrogenism (Rosenfield and Ehrmann, 2016; Ruth et al., 2020), insulin resistance (Diamanti-Kandarakis and Dunaif, 2012; Azziz et al., 2016), hyperinsulinemia (Housman and Reynolds, 2014; Muscogiuri et al., 2015; Cai et al., 2022), menstrual irregularity (Jayasena and Franks, 2014; Pena et al., 2020), anovulation (Dewailly et al., 2016; Carson and Kallen, 2021) or oligo-anovulation (Hickey et al., 2012; Tay et al., 2020), infertility (Carson and Kallen, 2021), and others. At the same time, PCOS significantly increases the risk of cardiovascular disease (Okoth et al., 2020; O'Kelly et al., 2022), type 2 diabetes (Diamanti-Kandarakis and Dunaif, 2012; Azziz et al., 2016; Zhu et al., 2021), obesity (Lim et al., 2012; Lim et al., 2013; Sermondade et al., 2019), and metabolic disorders (Rosenfield and Ehrmann, 2016; Escobar-Morreale, 2018).

Currently, oral contraceptives, antiandrogens, insulin sensitizers, and ovulation-stimulating drugs are mainly used to treat different symptoms of PCOS. However, these treatments only temporarily control symptoms, with a few side effects and no preventive effect. For example, oral contraceptives aggravate insulin resistance while increasing the risk of inflammation and coagulation disorders in women with PCOS (Manzoor et al., 2019; Okoth et al., 2020). Spironolactone, as a commonly used antiandrogen, plays an antagonistic role by binding to the androgen receptor despite potentially leading to menstrual irregularity and even feminizing male fetuses (Happe et al., 2021; Rashid et al., 2022). Although metformin is universally utilized as an insulin sensitizer in clinical practice, side effects (such as nausea, vomiting, and diarrhea) limit its extensive clinical application (Morley et al., 2017). For infertility caused by PCOS, assisted reproductive technology can be applied as an alternative method. However, ovulation-stimulating drugs may induce poor ovarian response and hyperstimulation syndrome, which limits the clinical application of this technology (La Marca and Sunkara, 2014). Therefore, it is urgent to discover drugs with novel mechanisms and few side effects to solve clinical problems.

**FIGURE 1:**
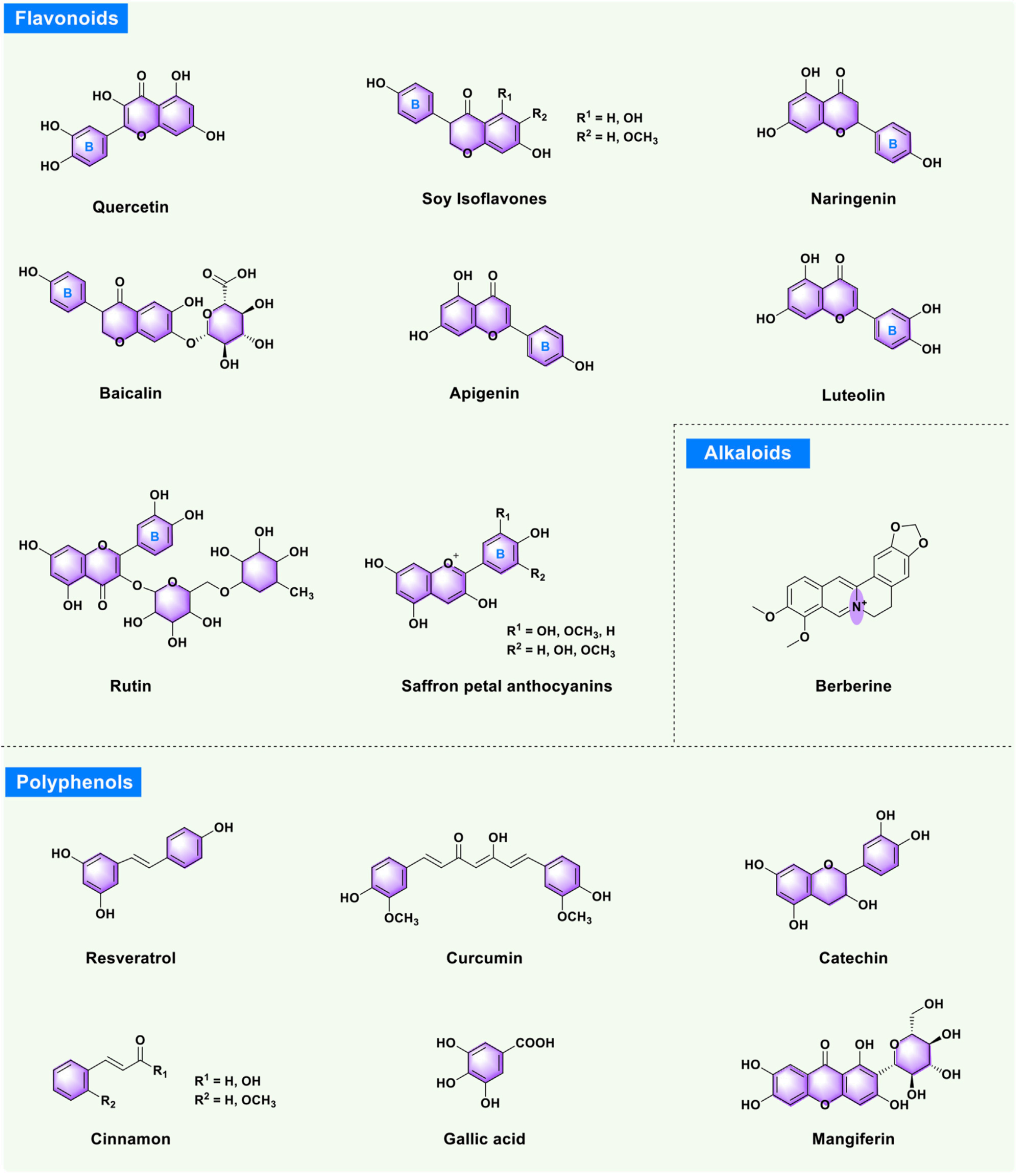
Bioactive phytochemicals isolated from TCM and medicinal plants forward PCOS.

**FIGURE 2:**
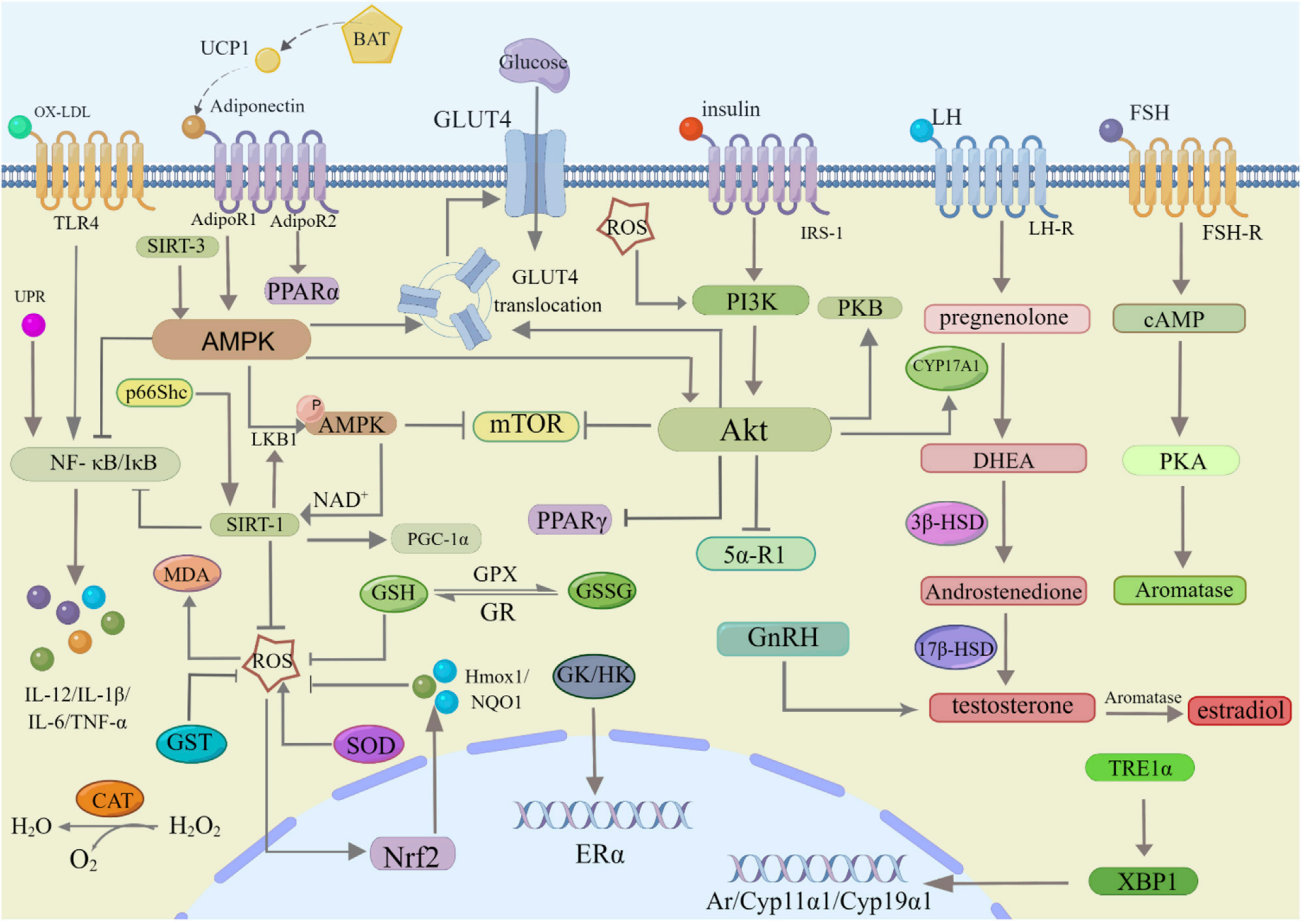
Therapeutic mechanisms of bioactive phytochemicals in PCOS.

Phytochemicals extracted from medicinal herbs and plants are a vital resource for discovering novel drugs (Choudhari et al., 2020; Rahman et al., 2021; Khatoon et al., 2022). In recent years, it has been found that phytochemicals isolated from traditional Chinese medicine (TCM) and medicinal plants (Figure 1) can improve symptoms of PCOS, such as oxidative stress, insulin resistance, hyperinsulinemia, hyperandrogenism, abnormalities in ovarian morphology and function, obesity, anovulation or oligomenorrhea, miscarriage, infertility, and others. Further mechanistic studies showed that these phytochemicals mainly prevent, alleviate, and treat PCOS symptoms through multiple mechanisms (Figure 2). Although the above research progress has laid a critical research foundation for the discovery of new drugs targeting PCOS, there is a lack of systematic reviews (despite the abundance of experimental literature and tiny specific-topic reviews (Mihanfar et al., 2021a) to summarize these advancements in this rapidly developing and promising field. In this context, this review systematically documents the progress of bioactive phytochemicals from TCM and plants in treating PCOS. For the convenience of readers, this review is divided into three parts (flavonoids, polyphenols, and alkaloids) according to the structural characteristics of these valuable phytochemicals.

## 2 The pathogenesis of polycystic ovary syndrome

Although the specific pathological mechanism of PCOS remains unclear, numerous studies have shown that oxidative stress (Murri et al., 2013), insulin resistance (Diamanti-Kandarakis and Dunaif, 2012), and androgen excess (Azziz et al., 2016; Rosenfield and Ehrmann, 2016) play an essential role in the occurrence and development of polycystic ovary syndrome. PCOS can be considered a state of oxidative stress (Merhi et al., 2019). Specifically, the antioxidant function in the human body cannot deal with excessive reactive oxygen species (ROS) (Sies and Jones, 2020; Cheung and Vousden, 2022), which further exacerbates the clinicopathologic features of PCOS, such as chronic oligo-anovulation or anovulation, clinical or biochemical signs of hyperandrogenism, and polycystic ovarian morphology in women. Meanwhile, high levels aggravate the oxidative stress reaction and are often accompanied by insulin resistance (Diamanti-Kandarakis and Dunaif, 2012), worsening PCOS symptoms. In addition, many factors, such as the maternal environment and genetics (Stener-Victorin and Deng, 2021), have influenced the evolution of PCOS. Namely, high prenatal maternal androgen levels cause PCOS in female infants (Filippou and Homburg, 2017). Moreover, recent studies disclose that multiple genes are highly related to PCOS, and data analysis shows that 241 gene mutations are involved in the etiology of PCOS (Joseph et al., 2016). These pathological factors indicate that PCOS is a comprehensive disease referring to multiple signaling pathways and targets.

Recent studies have found that the process of chronic inflammation plays an essential role in the pathogenesis of PCOS (Rudnicka et al., 2021). Numerous studies have shown that PCOS patients have higher inflammatory markers, such as TNF-α, IL-6, IL-1β, IL-18, and C-reactive protein (CRP) (Ganie et al., 2019; Liu et al., 2021). The nuclear factor κB (NF-κB) pathway and phosphatidylinositol 3-kinase (PI3K)/AKT serine (Akt) pathway are associated with high levels of inflammatory markers. It has been validated that the upregulation of these pathways is related to the mechanism of PCOS (Zhao et al., 2015). The higher activity of proinflammatory processes in adipocytes is related to insulin resistance (Zatterale et al., 2020). Therefore, obesity, inflammatory factors, and insulin resistance will jointly affect the occurrence and development of PCOS. Obesity is a symptom most PCOS patients face (de Zegher et al., 2018), and overweight and obese women have lower ovulation rates, conception rates, pregnancy rates, and live birth rates (Gao et al., 2021). Moreover, obesity can promote the molecular mechanism of androgen expression, which may be the cause of obesity leading to PCOS (Rangel et al., 2021). In addition, the latest studies have revealed that the gut microbiota is also connected with the pathogenesis of PCOS (Qi et al., 2019). Studies have shown that the abundance of *Firmicutes* and *Bacteroides* in the gut microbiota of PCOS patients is changed. As beneficial bacteria, *Lactobacilli* and *Bifidobacteria* can regulate the levels of sex hormones, manage the synthesis and secretion of insulin, and reduce the production of proinflammatory cytokines (He and Li, 2020; He et al., 2020; Yurtdas and Akdevelioglu, 2020). All these studies prove that restoring the abundance of some gut microbiota can be used as a new strategy to relieve PCOS symptoms.

## 3 Flavonoids

Flavonoids are a class of essential and valuable compounds frequently found in nature and demonstrate a variety of physiological activities, such as anti-inflammatory (Choy et al., 2019; Maleki et al., 2019), antioxidation (Shen et al., 2022), hypoglycemic (Shamsudin et al., 2022), antiviral (Ninfali et al., 2020; Badshah et al., 2021), and antitumor (Sun et al., 2022) activities. This part mainly summarizes the pharmacological effects of quercetin, soy isoflavones, naringenin, baicalin, apigenin, luteolin, rutin, and anthocyanins on PCOS (Table 1).

**TABLE 1:** Therapeutic effects of flavonoids on PCOS.

Compound	Detail	Cell lines/model	Dose	Application	Ref
Quercetin	Reduced the activity of steroidogenic enzyme 3β-HSD and/or 17β-HSD	Letrozole-induced PCOS rats model	25 mg/kg	*In vivo*	Hong et al. (2018)
	Inhibited PI3K pathway to decrease the expression of CYP17; Cyp17a1 gene	Testosterone propionate-induced PCOS rats model	150 mg/kg	*In vivo*	Shah and Patel, (2016)
	Affected the combination of AR with specific sequences of CNP and NPR2 gene promoters	DHEA-induced PCOS rats model	100 mg/kg	*In vivo*	(Zheng et al., 2022)
			4 mg/kg (intraperitoneal injection)		
	Reduced the expression of aromatase and the estrogen level and increased the expression of Nesfatin-1 and AdipoR1 genes	DHEA-induced PCOS rats model	15 mg/kg	*In vivo*	Khorchani et al. (2020)
	Restored the activities of HK and GK in the liver and increased the GLUT4 and the ERα gene	DHEA-induced PCOS rats model	15 mg/kg (0.5 ml 10% ethanol)	*In vivo*	Neisy et al. (2019)
	Activated the adiponectin pathway and AMPK in PBMC	Women with PCOS	1000 mg	*clinical study*	Rezvan et al. (2018)
	Activated the expression of AMPK-SIRT-1 protein	Letrozole-induced PCOS rats model	100 mg/kg (CMC 0.5%)	*In vivo*	Mihanfar et al. (2021b)
	Enhanced the levels of SOD, CAT, GPX, GST, GSH, and GR	Menopausal female Sprague‒Dawley (SD) rats	12.5, 25, 50 mg/kg	*In vivo*	*(Wang et al., 2018)*
		Immature female SD rats’ granulosa cells	5, 20, 50 μM	*In vitro*	
	Regulated the OX-LDL/TLR-4/NF-κB pathway and reduced the levels of inflammatory cytokines IL-1β, IL-6, and TNF-α	DHEA-induced PCOS rats model	100 mg/kg	*In vivo*	(Wang et al., 2017)
Soy Isoflavones	Reduced the TOS and inflammatory cytokines IL-6, IL-12, IL-1β, TNF-α and increased the TAC	EV-induced PCOS rats model	50, 100 mg/kg	*In vivo*	Farkhad and Khazali, (2019)
	Inhibited NF-κB pathway	Letrozole-induced PCOS rats model	100 mg/kg	*In vivo*	Ma et al. (2021)
	Increased aromatase activity to decrease the level of testosterone	Letrozole-induced PCOS rats model	50, 100 mg/kg	*In vivo*	Rajan et al. (2017)
	Reduced the BMI, blood glucose, insulin resistance markers (such as insulin), total testosterone in serum, SHBG, FAI, triglyceride, VLDL-cholestero, and MDA	Participants with PCOS aged 18–40 years	50 mg	*clinical study*	Jamilian and Asemi, (2016)
Naringenin	Reduced the activities of steroidogenic enzyme 3β-HSD and 17β-HSD	Letrozole-induced PCOS rats model	20 mg/kg	*In vivo*	Hong et al. (2019)
	Activating the AMPK/SIRT-1/PGC-1α signal pathway and regulating the composition of gut microbiota	Letrozole-induced PCOS rats model	20 mg/kg	*In vivo*	(Wu et al., 2022)
	Blocked the mTORC1/mTORC2 signal	Insulin + hCG-induced PCOS rats model	200 mg/kg (CMS, 1% w/v)	*In vivo*	(Yang et al., 2022)
Baicalin	Destroyed the binding of the HSD3B2 gene to promoter GATA1 and reduced the expression of ovarian HSD3B2	NCI-H295R cells	25 μmol/L, 24 h	*In vivo*	(Yu et al., 2019)
		DHEA-induced PCOS rats model	20 mg/kg (normal saline)		
	Increased AMPK to inhibit PI3K/Akt signal pathway and reduce the protein level of 5α-R1	DHEA-induced PCOS rats model	50 mg/kg (injected)	*In vivo*	(Wang et al., 2019)
Apigenin	Reduced levels of testosterone, estrogen, and LH/FSH Reduced the expression of the NF-κB transcription factor gene to reduce the levels of TNF-α and IL-6	EV-induced PCOS rats model	20, 40 mg/kg (1 mg/ml in DMSO)	*In vivo*	Darabi et al. (2020)
	Decrease the TOS and increase the TAC and the activities of SOD, CAT, POD, NADPH, and GR	DHEA-induced PCOS rats model	20 mg/kg (0.5% carboxymethyl cellulose)	*In vivo*	Peng et al. (2022)
Luteolin	Regulated PI3K/Akt transduction	Letrozole-induced PCOS rats model	25, 50, 100 mg/kg (intraperitoneal injection)	*In vivo*	Huang and Zhang, (2021)
	Activated Nrf2 to promote the expression of Hmox1 and NQO1				
Rutin	Enhanced the activity of insulin-dependent receptor kinase and increases the expression of GLUT4	Letrozole-induced PCOS rats model	100, 150 mg/kg	*In vivo*	Jahan et al. (2016)
	Activated BAT to increase the expression of UCP1 and upregulates the expression of adiponectin	DHEA-induced PCOS rats model	100 mg/kg	*In vivo*	Hu et al. (2017)
	Reduced the expression of GnRH and the secretion of LH and testosterone	5α-DHT-induced PCOS rats model	150, 300 mg/kg (intraperitoneal injection)	*In vivo*	Gao et al. (2020)
Saffron petal anthocyanins	Restored the levels of LH, FSH, and steroid hormones	Testosterone enanthate-induced PCOS mice model	20, 40, 80 mg/kg	*In vivo*	Moshfegh et al. (2022)
	Reduced the mRNA levels of inflammatory genes and restored the NF-κB and IκB				

### 3.1 Quercetin

Quercetin (3,5,7,3′,4′-pentahydroxyflavone) is a common dietary flavonoid in vegetables and fruits. In recent years, quercetin has been widely studied because of its antioxidation, anti-inflammatory, antitumor, hypoglycemic, cardiovascular protection, and other effects in regulating ovarian function and protecting ovarian morphology. Through network pharmacology and bioinformatics, Li et al. found that quercetin, as the core ingredient of the Kuntai capsule (a proprietary Chinese medicine), overlaps with potential therapeutic targets in treating PCOS (Li et al., 2022). Hong et al. speculated that the phenolic ring B of quercetin reduced the activity of steroidogenic enzyme 3β-hydroxysteroid dehydrogenase (3β-HSD) and/or 17β-hydroxysteroid dehydrogenase (17β-HSD), thus effectively relieving the symptoms of the metabolic disorder (Hong et al., 2018). In addition, quercetin decreases the expression of the 17α-hydroxylase/C17-20-lyase (CYP17; Cyp17a1) gene to reduce the activity of its enzyme (the critical enzyme that converts progesterone to androgens) (Shah and Patel, 2016) and regulates the state of androgen receptor (AR) binding to the specific sequence of C-type natriuretic peptide (CNP)/natriuretic peptide receptor 2 (NPR2) gene promoters (Zheng et al., 2022), thereby restoring the normal meiosis of oocytes and improving the state of high androgen and anovulation in patients with PCOS. In addition to high androgen levels, Khorchani et al. found that the overexpression of aromatase is associated with an increase in estrogen levels. In contrast, estrogen inhibits the secretion of gonadotropin-releasing hormone (GnRH) and follicle-stimulating hormone (FSH) through negative feedback, leading to decreased oogenesis (Khorchani et al., 2020). Quercetin, functionally similar to estradiol, can reduce the expression of aromatase and the estrogen level, effectively increasing oogenesis (Khorchani et al., 2020). Estrogen receptor α (ERα) plays a vital role in fertility. Neisy et al. found that the ERα gene can be increased by quercetin. Compared with the control group, the expression of the ERα gene reached five times higher in the rats treated with quercetin, which provided a method for treating infertility caused by PCOS (Neisy et al., 2019).

Obesity is one of the crucial factors attributed to PCOS. Khorchani et al. reported that low expression of the Nesfatin-1 and AdipoR1 genes was associated with obesity, and treatment with quercetin increased the expression of the Nesfatin-1 and AdipoR1 genes, showing a good effect on weight loss and other symptoms caused by obesity (Khorchani et al., 2020). Adiponectin is an essential human adipose factor controlled by the receptors AdipoR1 and AdipoR2, which can metabolize glucose and fatty acids by stimulating AMP-activated protein kinase (AMPK) (Rezvan et al., 2018). The level of adiponectin is low in PCOS with or without adiposity. Clinical data showed that quercetin (1 g daily for 12 weeks) improved adiponectin-mediated IR and metabolic disorder by increasing total adiponectin and high molecular weight adiponectin and reducing resistin, which promoted IR and hyperinsulinemia (Khorshidi et al., 2018). Furthermore, Rezvan et al. revealed that by activating the adiponectin pathway and AMPK in peripheral blood mononuclear cells (PBMCs), quercetin improved the metabolic pathway and the expression of adipokines (Rezvan et al., 2018). Moreover, the imbalance of AMPK-SIRT-1 (as a metabolic sensor) is highly related to IR, weight gain, and fat formation. Mihanfar et al. reported that quercetin could activate the expression of AMPK-SIRT-1 protein, thus increasing insulin sensitivity and improving other symptoms of PCOS (Mihanfar et al., 2021b).

Oxidative stress is another prominent pathological feature of PCOS. Research has found that quercetin enhances the levels of superoxide dismutase (SOD), catalase (CAT), glutathione peroxidase (GPX), glutathione s-transferase (GST), glutathione (GSH), and glutathione reductase (GR) in menopausal female Sprague‒Dawley (SD) rats to reverse the changes in ovarian morphology induced by oxidative stress (Hong et al., 2018; Wang et al., 2018). In addition, quercetin also has promising potential to alleviate IR. Neisy et al. discovered that quercetin increased insulin sensitivity and restored glucose homeostasis by restoring the activities of hexokinase (HK) and glucokinase (GK) in the liver and the expression of the intrauterine glucose transporter type 4 (GLUT4) gene (Neisy et al., 2019). Later, Wang et al. found that quercetin reduced the levels of the inflammatory cytokines IL-1β, IL-6, and TNF-α by regulating the oxidized low-density lipoprotein (OX-LDL)/Toll-like receptor 4 (TLR-4)/nuclear factor κB (NF-κB) pathway, improving the inflammatory microenvironment of ovarian tissue and reversing IR in dehydroepiandrosterone (DHEA)-induced PCOS rats (Wang et al., 2017).

### 3.2 Soy isoflavones

Soy isoflavones are a kind of secondary metabolite formed in the growth of soy and display diverse bioactivities. In recent years, soybean isoflavones have attracted much attention due to their antioxidation, anti-inflammatory, and cholesterol-lowering properties. Farkhad et al. found that treatment with soy isoflavones could reduce the total oxidative state (TOS) and inflammatory cytokines IL-6, IL-12, IL-1β, and TNF-α in estradiol valerate (EV)-induced PCOS rats and increase the total antioxidant capacity (TAC) (Farkhad and Khazali, 2019). Their report revealed that soy isoflavones reduced the number of cystic follicles and the outer layer of theca cells (Lan et al., 2017). In addition, Ma et al. found that soy isoflavones regulated the morphology and function of ovaries by inhibiting the NF-κB pathway to achieve antioxidation and anti-inflammatory effects, improving hormone disorders in PCOS rats (Ma et al., 2021). Aromatase is a critical enzyme in converting androgen to estrogen, and insufficient aromatase activity may contribute to excessive androgen accumulation. Soy isoflavone decreased testosterone concentration by increasing aromatase activity and improving hyperandrogenism in letrozole-induced PCOS rats (Rajan et al., 2017). Clinical data studies showed that soy isoflavones (50 mg daily for 12 weeks), a reduction in BMI, blood glucose, insulin resistance markers (such as insulin), total serum testosterone, sex hormone-binding globulin (SHBG), free androgen index, triglyceride, VLDL-cholesterol, and malondialdehyde were observed, as well as increases in GSH, SOD, and GPX (Jamilian and Asemi, 2016; Karamali et al., 2018). These experiments indicated that soy isoflavones have beneficial effects on IR, hormone disorders, and oxidative stress related to PCOS. For gut microbiota, Liyanage et al. have found that letrozole-induced PCOS rats with soy isoflavones and resistant starch can restore the menstrual cycle and improve the polycystic ovary through the genus-level of gut microbiota returned to normal (Liyanage et al., 2021). However, the poor effect of soy isoflavone used exclusively was documented, which implied that soy isoflavone could be applied as an auxiliary drug to regulate gut microbiota (Liyanage et al., 2021). In contrast to the above studies, Patisaul et al. have shown that lifetime exposure to a soy diet containing endocrine-active phytoestrogens could induce the symptoms of PCOS caused by endocrine-disrupting compounds (EDCs) (Patisaul et al., 2014). Therefore, attention should be given to this phenomenon in treating PCOS.

### 3.3 Naringenin

Naringin, one of the valuable flavonoids widely found in traditional Chinese medicine, possesses a variety of pharmacological activities, such as anti-inflammation and antioxidation. Due to the existing B-ring moiety, naringin can reduce the activities of steroidogenic enzymes 3β-HSD and 17β-HSD in the letrozole-induced PCOS rats and improve the symptoms of hormone disorder (Hong et al., 2019). Additionally, naringin can enhance the bioactivities of SOD, CAT, and GPX while scavenging ROS and relieving the oxidative stress of PCOS (Hong et al., 2019). Wu et al. found that naringin could affect the level of sex hormones, reduce IR, alleviate chronic inflammation and maintain normal ovarian morphology and function by activating the AMPK/SIRT-1/PGC-1α signaling pathway and regulating the composition of intestinal flora (Wu et al., 2022). Furthermore, Yang et al. disclosed that naringin combined with morin could inhibit the proliferation of human endometrial adenocarcinoma cells. In their study, naringin inhibited PCOS-induced endometrial hyperplasia and regulated autophagy by blocking the mammalian target of rapamycin complex 1 (mTORC1)/mammalian target of rapamycin complex 2 (mTORC2) signaling, and morin increased the levels of Caspase-3 and LC3-phosphatidylethanolamine conjugate (LC3-II) (Yang et al., 2022). In addition, naringin could change the elevated serum insulin level and maintain the typical morphology of the ovary in rats with PCOS (Wu et al., 2022). Undoubtedly, naringin shows specific therapeutic potential in insulin-resistant PCOS.

### 3.4 Baicalin

*Scutellaria baicalensis* Georgi has been used for thousands of years in Asia. As an effective flavonoid isolated from *Scutellaria baicalensis* Georgi, baicalin has a wide range of pharmacological activities, such as anti-inflammatory, antitumor, and antioxidation activities. Overexpression of 3β-hydroxysteroid dehydrogenase type II (HSD3B2) in ovarian tissue or adrenal cortex leads to excessive androgen synthesis and results in PCOS (Yu et al., 2019). In NCI-H295R cells and the DHEA-induced PCOS rat model, Yu et al. discovered that baicalin could improve the hyperandrogenism of PCOS by destroying the binding of the HSD3B2 gene to the promoter GATA1 and reducing the expression of ovarian HSD3B2, resulting in reduced testosterone secretion (Yu et al., 2019). Additionally, previous studies have shown that AMPK, a sensor of cellular energy changes and a key enzyme in regulating glucose and lipid metabolism, participates in the synthesis and oxidative decomposition of glucose and fatty acids (You et al., 2018). Wang et al. reported that baicalin increased AMPK to inhibit the PI3K/Akt signaling pathway and reduce the protein level of 5α-R1 in ovarian tissue, contributing to increasing insulin sensitivity and regulating androgen levels (reducing the hormone levels of free testosterone, total testosterone, luteinizing hormone (LH), FSH, progesterone, and estradiol). Likewise, baicalin decreased TNF-α, IL-1β, and IL-18 and increased IL-10, resulting in the amelioration of inflammation by proinflammatory and anti-inflammatory cytokines in ovarian tissues (Wang et al., 2019).

### 3.5 Apigenin

Apigenin is a natural flavonoid from many sources, such as fruits and vegetables, with anti-inflammatory, antioxidation, and antitumor activities. Darabi et al. revealed that apigenin could reduce testosterone, estrogen, and LH/FSH levels in EV-induced PCOS rats, normalizing the hormone levels (Darabi et al., 2020). Moreover, they speculated that the observed decrease in hormone levels might be achieved by reducing the activity of aromatase and its 17β-hydroxysteroid dehydrogenases. PCOS is a chronic inflammatory disease highly related to oxidative stress. The experiment showed that apigenin reduced TNF-α and IL-6, which may be accomplished by reducing the expression of the NF-κB transcription factor gene (Darabi et al., 2020). Peng et al. found that apigenin could decrease the TOS and increase the TAC and the activities of SOD, CAT, peroxidase (POD), nicotinamide adenine dinucleotide phosphate (NADPH), and glutathione reductase (GR), thus inhibiting oxidative stress (Peng et al., 2022). As a result, the antioxidant effect of apigenin restored ovarian tissue, increased the number of healthy follicles, and contributed to regaining the pleura of follicles (Darabi et al., 2020; Peng et al., 2022).

### 3.6 Luteolin

Luteolin is a natural flavonoid in fruits, vegetables, and TCM used to treat hypertension, diabetes, cancer, and allergies. It is generally accepted that IR, which is closely related to hyperinsulinemia, is one of the pathogenic mechanisms of PCOS. The abundance of insulin receptor substrate (IRS) is related to decreased PI3K activity in women with PCOS (Chahal et al., 2021). The phosphorylation of IRS in PCOS results in the dysfunction of PI3K/Akt transduction, eventually leading to exaggerated IR and disturbance of glucose homeostasis. Luteolin showed significant glucose homeostasis recovery and IR improvement in letrozole-induced PCOS rats by regulating PI3K/Akt transduction (Huang and Zhang, 2021). Additionally, PCOS patients always showed a state of oxidative stress. The decrease in antioxidants, such as SOD, CAT, GSH, GPX, and glutathione peroxidase (GSH-Px), and the increase in ROS affected the ovary’s physiological functions, such as ovulation, folliculogenesis, and oocyte maturation. Moreover, Nrf2 is a transcription factor mediating the antioxidant pathway. Huang et al. reported that luteolin exerted antioxidative effects by activating Nrf2 to promote the expression of heme oxygenase-1 (Hmox1) and quinone oxidoreductase 1 (NQO1), as well as increasing the levels of CAT, GPX, SOD, GSH, and GSH-Px. As a result, reduced oxidative stress and restored ovarian function were observed in ovarian granulosa cells of rats with letrozole-induced PCOS (Huang and Zhang, 2021).

### 3.7 Rutin

Rutin is a flavonol compound from many sources with antioxidative, anti-inflammatory, and hypoglycemic effects. Dyslipidemia, IR, hormonal imbalances, and oxidative stress often exist in PCOS. Rutin can restore lipid profiles by reducing the expression levels of adipogenic genes (Han et al., 2016). For IR, rutin could enhance the activity of insulin-dependent receptor kinase and increase the expression of GLUT4, thus increasing glucose uptake, controlling blood sugar, and reducing the risk of diabetes in patients with PCOS (Jahan et al., 2016). The function and morphology of aberrant adipose tissue in PCOS patients are closely related to IR. BAT (brown adipose tissue) can participate in whole-body metabolic homeostasis through uncoupling protein 1 (UCP1)-mediated thermogenesis and secretory cytokines such as adiponectin. Previous research experimentally showed that BAT transplantation in DHEA-induced PCOS rats improved lipid metabolism and reversed anovulation, hyperandrogenism, and polycystic ovaries (Yuan et al., 2016). However, BAT transplantation is unfeasible in clinical applications. Moreover, rutin could activate BAT, increase the expression of UCP1 to increase energy expenditure, and upregulate adiponectin expression in BAT, thereby improving adiposity and IR in DHEA-induced PCOS rats (Hu et al., 2017). Treatment with rutin activated ovarian steroidogenic enzymes such as P450C17, aromatase, 3β-HSD, 17β-HSD, and STAR at normal levels and improved the hormonal imbalances of PCOS rats (Hu et al., 2017). Furthermore, rutin reduced ROS accumulation and improved oxidative stress in PCOS rats. As a rutin derivative, troxerutin was capable of reducing the expression of GnRH (as the master hormone), reducing the secretion of LH and testosterone, and improving the reproductive endocrine dysfunction of 5α-DHT-induced PCOS rats (Gao et al., 2020).

### 3.8 Saffron petal anthocyanins

Crocus sativus (saffron) possesses anti-inflammation, antioxidation, and antitumor properties in the clinical application of TCM. Anthocyanins belong to the flavonoid family and are used to alleviate diabetes and control obesity, which contribute to PCOS. Moshfegh et al. utilized saffron petal anthocyanins (SPA) to treat testosterone-induced PCOS mice and disclosed that SPA could improve the hormone disorder, which was evidenced by the recovery of serum levels of LH, FSH, and steroid hormones (estrogen, progesterone, and testosterone) in mice (Moshfegh et al., 2022). In addition, SPA reduced the levels of the inflammatory cytokines TNF-α, IL-6, IL-1β, IL-18, and C-reactive protein (CRP), indicating that SPA demonstrated an anti-inflammatory effect by reducing the mRNA levels of inflammatory genes and restoring NF-κB and inhibitor of NF-κB (IκB) (key mediators of inflammatory genes), resulting in improvement of the inflammatory response of PCOS mice (Moshfegh et al., 2022). In addition, SPA showed antioxidation, which was reflected in the increased levels of GPX, SOD, CAT, GST, and GSH enzymes in the plasma of PCOS mice after SPA treatment. Subsequently, these results ensured that the number of follicles and corpus luteum increased, and the number of cystic follicles decreased in PCOS mice (Moshfegh et al., 2022).

## 4 Polyphenols

Polyphenols play a restricted role in modern medicine because of their poor bioavailability (Dei Cas and Ghidoni, 2019; Zhang et al., 2020a). However, polyphenols possess significantly beneficial effects in treating PCOS, which has been confirmed in recent studies (Mirabelli et al., 2020; Mihanfar et al., 2021a). This section mainly focuses on the pharmacological effects of polyphenols such as resveratrol, curcumin, catechins, cinnamon, gallic acid, and mangiferin on PCOS (Table 2).

**TABLE 2:** Therapeutic effects of polyphenols on PCOS.

Compound	Detail	Cell lines/model	Dose	Application	Ref
Resveratrol	Reduced the expression of VEGF and HIF1 genes	women with PCOS	800 mg	*clinical study*	Bahramrezaie et al. (2019)
	Decreased VEGF in granulosa cells	Granulosa cells	10–50 μM	*In vitro*	Ortega et al. (2012b)
	Reduced levels of testosterone and DHEA	Women with PCOS	1500 mg	*clinical study*	Banaszewska et al. (2016)
	Inhibited the Akt/PKB signal pathway	NCI-H295R cells	5–50 μM (DMSO)	*In vitro*	Marti et al. (2017)
	Blocked the activity of the Akt/PKB signaling pathway to inhibit Cyp17a1 mRNA expression	Ovarian the interstitial cells	1–10 μM	*In vitro*	Ortega et al. (2012a)
	Inhibited the mevalonate pathway and Akt/PKB phosphorylation	Ovarian the interstitial cells	3–10 μM	*In vitro*	Ortega et al. (2014)
	Mediated deacetylation of the p66Shc and reduced the production of ROS and fibrotic factors	DHEA-induced PCOS rats model	100 mg/kg	*In vivo*	*(Wang et al., 2020)*
	Altered the expression of genes involved in the UPR process	Women with PCOS	800 mg	*clinical study*	Brenjian et al., 2020
	Inhibited NF-κB and NF-κB regulated gene products				
	Activated the AMPK-SIRT-1 pathway and reduced the levels of AMH, testosterone, LH, and LH/FSH	DHEA-induced PCOS rats model	20 mg/kg	*In vivo*	*Rencber et al., 2018*
	Resumed the inward flow of calcium ions, activated the CaMKIIβ, and enabled TZPs synthesis	TBT-induced PCOS rats model	20 mg/kg (0.5% CMC)	*In vivo*	Chen et al. (2022)
Curcumin	Inhibited the IRE1α-XBP1 pathway in ovarian GCs, and downregulated follicular development-related genes (Ar, Cyp11α1, and Cyp19α1)	DHT-induced PCOS rats model	200 mg/kg	*In vivo*	(Zhang et al., 2021)
	Regulated the level of PI3K/Akt/mTOR in the pancreas and reduce the level of TNF-α	Letrozole-induced PCOS rats model	50, 100, 200 mg/kg	*In vivo*	Abuelezz et al. (2020)
	Reduced the expression of apoptotic factors and increased the expression of BAX, Bcl2 genes, and Caspase3	DHEA-induced PCOS mice model	5.4 mg/100 g (54 mg/kg)	*In vivo*	Abhari et al. (2020)
	Achieve a natural drug delivery system (sustained release pattern) against PCOS for a long time	KGN cells	5 μg/ml	*In vitro*	Raja et al. (2021)
		EV-induced PCOS mice model	50 mg/kg	*In vivo*	
	Reduced FPG and DHEA	Women with PCOS	1500 mg	*clinical study*	Heshmati et al. (2021)
Catechin	Inhibited the expression of STAT3 signaling, MMP2, and MMP9 in the uterus, increased IRS-1 and PI3K signals, downregulated NF-κB	Insulin and hCG- induced PCOS mice model	25, 50, 100 mg/kg	*In vivo*	Hong et al. (2020)
	Activated PPAR-α, and PPAR-γ	Woman with PCOS	200 mg	*clinical study*	Haj-Husein et al. (2016)
Cinnamon	Enhanced insulin sensitivity and reduced the levels of insulin and LDL	Woman with PCOS	1500 mg	*clinical study*	Hajimonfarednejad et al. (2018)
	Reduced the plasma IGF-1 level, increased the plasma IGFBP-1 level, and downregulated the serum levels of testosterone and insulin	DHEA-induced PCOS mice model	10 mg/100 g	*In vivo*	Dou et al. (2018)
			(100 mg/kg)		
Gallic acid	Decreased the concentrations of inflammatory cytokines and inhibited ROS production	EV-induced PCOS rats model	50, 100 mg/kg	*In vivo*	Mazloom et al. (2019)
Mangiferin	Reduced the pp65/p65 ratio and blocked the NF-κB signaling and the expression of inflammatory cytokines IL-6, IL-1β, and TNF-α	DHEA-induced PCOS rats model	10, 20, 30 mg/kg	*In vivo*	Qian et al. (2020)

### 4.1 Resveratrol

PCOS often presents with hyperandrogenism, oxidative stress, and infertility, accompanied by cardiovascular diseases and others. Resveratrol (RVT) is a polyphenolic compound derived from peanuts, grapes, and other plants featuring broad biological functions, such as antioxidation, anti-inflammatory, and cardiovascular protective effects.

Hyperandrogenism in PCOS induces the expression of VEGF (highly related to ovarian hyperstimulation syndrome) and leads to abnormal angiogenic irregularities in ovaries (Herr et al., 2015). Ortega and Bahramrezaie et al. illustrated that RVT improved irregular angiogenesis by reducing the expression of VEGF mRNA, VEGF protein, and the intermediate factor HIF1 to improve disorders of ovulation, subfertility, and endometriosis caused by ovarian hyperstimulation syndrome (Ortega et al., 2012b; Bahramrezaie et al., 2019). The high testosterone secreted by the ovary and high DHEA secreted by the adrenal gland could induce hyperandrogenism. Banaszewska et al. found that RVT reduced the levels of testosterone and DHEA in serum and affected the production of androgen in the ovary and adrenal glands, thus improving hyperandrogenism in PCOS (Banaszewska et al., 2016). Further study showed that CYP17/Cyp17a1 was the key rate-limiting enzyme in androgen biosynthesis. RVT reduced androgen production by inhibiting the Akt/PKB signaling pathway and reducing the protein expression of CYP17/Cyp17a1 in theca-interstitial cells (Ortega et al., 2012a; Marti et al., 2017). Moreover, Israel et al. confirmed that RVT, combined with simvastatin (a drug that inhibits steroidogenesis in theca-interstitial cells), could effectively reduce steroidogenesis by inhibiting the mevalonate pathway and Akt/PKB phosphorylation (Ortega et al., 2014).

Hyperandrogenism also induces ovarian oxidative stress (OS) and fibrosis in PCOS rats, leading to infertility. Wang et al. reported that RVT, as a SIRT-1 agonist, mediated deacetylation of the 66-kDa Src homology 2 domain-containing protein (p66Shc) while reducing the production of ROS and fibrotic factors, resulting in improved ovarian oxidative stress and fibrosis (Wang et al., 2020). Endoplasmic reticulum stress (ERS) in granulosa cells (GCs) is related to chronic inflammation and oxidative stress in PCOS. Studies have shown that RVT can regulate ERS in GCs by altering the expression of genes involved in the unfolded protein response (UPR) process, reducing the levels of proinflammatory factors, such as IL-6, IL-1β, TNF-α, IL-18, and CPR, and contributing to the amelioration of inflammation and oxidative stress (Ghowsi et al., 2018; Brenjian et al., 2020). Rencber et al. further disclosed that RVT could not only reduce the level of these factors by activating the AMPK-SIRT-1 pathway but also downregulate the levels of anti-Müllerian hormone (AMH), T, LH, and LH/FSH, giving rise to ameliorate the hormone disorders of PCOS and the structure of follicular cells (Rencber et al., 2018). Recent studies have pointed out that damage to transzonal projections (TZPs) could mediate oocyte-granulosa cell (GC) communication in follicles, which may play a vital role in the etiology of PCOS. Chen et al. disclosed that RVT resumed the inward flow of calcium ions and activated calcium-/calmodulin-dependent protein kinase II beta (CaMKIIβ), thus enabling the synthesis of TZPs. Chen’s work provided a new strategy for treating PCOS (Chen et al., 2022).

### 4.2 Curcumin

Curcumin is a yellow polyphenol pigment extracted from turmeric (*Curcuma longa* L.), which is low-insoluble in water. Curcumin has received attention from the scientific community due to its anti-inflammatory, antioxidation, and antiapoptotic properties. In PCOS, hyperandrogenism induces ERs and activation of the UPR (to maintain ER homeostasis). Zhang et al. illustrated that long-term ERs led to GC autophagy or apoptosis and caused follicle development disorders, including the accumulation of small follicles around the ovary, polycystic morphology, anovulation, and damage to follicular maturation (Zhang et al., 2021). Curcumin (200 mg/kg daily for 8 weeks) combined with aerobic exercise ameliorated follicle development disorders by reducing hyperandrogen-induced ERs, inhibiting the IRE1α-XBP1 pathway in ovarian GCs, and downregulating follicular development-related genes (Ar, Cyp11α1, and Cyp19α1) (Zhang et al., 2021). Heshmati et al. found that in a randomized, placebo-controlled double-blind trial, curcumin (1500 mg daily for 12 weeks) significantly reduced fasting plasma glucose (FPG) and DHEA in PCOS patients. This result suggested that curcumin could reduce blood glucose, regulate hormone levels, and prevent type 2 diabetes and other complications with PCOS (Heshmati et al., 2021). Due to curcumin’s low oral bioavailability, medium-high curcumin was utilized in the above experiments.

To improve curcumin’s solubility and bioavailability, many researchers have applied curcumin nanoparticles (curcumin NPs) to improve its therapeutic effects. Abuelezz et al. reported that impaired PI3k/Akt/mammalian target of rapamycin (mTOR) signaling in the pancreas and increased levels of TNF-α in PCOS led to pancreatic β-cell secretory dysfunction and IR to disturb glucose metabolism. Curcumin NPs, as an anti-inflammatory agent, could regulate the level of PI3K/Akt/mTOR in the pancreas and reduce TNF-α, thus alleviating abnormal glucose metabolism (Abuelezz et al., 2020). In addition, Abhari et al. revealed that the imbalance between increased ROS levels and decreased GSH initiates oxidative stress and induces apoptosis (Abhari et al., 2020). Oxidative stress could increase the levels of AMH and estrogens *via* lipid peroxidation of GCs and then bring about follicular atresia, polycystic morphology, anovulation, and other symptoms in PCOS. Interestingly, curcumin’s antioxidation could reduce the level of ROS and inhibit GC apoptosis and oxidative stress by reducing the expression of apoptotic factors and increasing the expression of BAX, B-cell lymphoma 2 (Bcl2) genes, and Caspase3, followed by restoring ovarian function (Abhari et al., 2020). Inspired by the above studies, Raja et al. modified curcumin NPs, which encapsulated arginine (Arg)- and N-acetyl histidine (NAcHis)-modified chitosan (Arg-CS-NAcHis/Cur) nanoparticles (NPs), to construct a better natural drug delivery system (sustained release pattern) against PCOS (Raja et al., 2021).

### 4.3 Catechin

Bioactive ingredients extracted from tea potentially improve IR, hyperandrogenism, abnormalities in ovarian morphology, and overweight in PCOS through antioxidation and anti-inflammatory pathways. Catechins from oolong tea inhibited the expression of STAT3 signaling, MMP2, and MMP9 in the uterus, increased insulin receptor substrate-1 (IRS-1) and PI3K signals, and downregulated NF-κB, contributing to ameliorating hyperandrogenism and IR, reversing abnormalities in ovarian morphology and reducing uterine inflammation in insulin- and hCG-induced PCOS mice (Hong et al., 2020). The phenolic content in marjoram (*Origanum majorana* L.) tea aqueous extract mainly includes caffeic acid derivatives and rosmarinic acid. Haj-Husein et al. illustrated that marjoram tea increased PCOS-induced insulin sensitivity by activating peroxisome proliferator-activated receptor-α (PPAR-α) and peroxisome proliferator-activated receptor-γ (PPAR-γ) (Haj-Husein et al., 2016). A host of PCOS patients are overweight or obese, which will not only affect metabolism but also lead to reproductive disorders. Mombaini et al. demonstrated that green tea reduced body mass index, weight, waist circumference, and body fat percentage in women with PCOS and revealed that catechins and caffeine in green tea could prolong the pharmacological effects of catecholamines and increase levels of norepinephrine (increasing energy expenditure and fat oxidation) (Mombaini et al., 2017). These experiments suggested that long-term tea consumption was beneficial in preventing PCOS.

### 4.4 Cinnamon

Cinnamon polyphenol compounds isolated from cinnamon have attracted much attention because of their potential in patients with type 2 diabetes. Kort et al. showed that cinnamon could significantly reduce FPG and improve IR and menstrual cyclicity in patients with PCOS, but the specific mechanism has not been clarified (Kort and Lobo, 2014). Further mechanistic studies showed that cinnamon extract had insulin-like properties, enhancing insulin sensitivity and reducing insulin levels and LDL. As a result, the improvement of hormone levels, recovety of the normal menstrual cycle, and reduction of the risk of PCOS complications such as type 2 diabetes and cardiovascular disease were observed (Keefe, 2015; Hajimonfarednejad et al., 2018; Khan and Begum, 2019). In addition, the imbalance between insulin-like growth factor-1 (IGF-1) and insulin-like growth factor-binding protein-1 (IGFBP-1) affects follicular maturation. Patients with PCOS always feature higher IGF-1 and lower IGFBP-1 than healthy women. Dou et al. found that cinnamon extract could reduce the plasma IGF-1 level, increase the plasma IGFBP-1 level, and downregulate the serum levels of testosterone and insulin, followed by restoring the estrous cyclicity and ovary morphology of DHEA-induced PCOS mice (Dou et al., 2018).

### 4.5 Gallic acid

Gallic acid (GA), with the chemical formula C_7_H_6_O_5_ and a molecular weight of 170.12 g/mol, is a polyphenol found in many plants with anti-inflammatory and antioxidation properties. Mazloom et al. showed that proinflammatory cytokines were significantly increased, and estrous cyclicity was disrupted in the EV-induced PCOS rat model (Mazloom et al., 2019). The decreased concentrations of inflammatory cytokines in the ovaries of rats treated with GA indicated that GA played an anti-inflammatory role in PCOS (Mazloom et al., 2019). As an antioxidant, GA could effectively protect the ovary and improve the blocked development of follicular formation and follicular atresia in PCOS by inhibiting ROS production, protecting DNA, and preventing lipid peroxidation (Mazloom et al., 2019).

### 4.6 Mangiferin

Mangiferin is a bioactive compound isolated from the leaves and bark of mango tree (Mangifera indica) and kinds of TCM, which is widely reported due to its anti-inflammatory and regulation of blood glucos. Qian et al. demonstrated that mangiferin could ameliorate ovarian function and IR by reducing the ratio of ovarian weight/body weight, levels of blood glucose, and insulin, which was highly connected with POCS (Qian et al., 2020). In Qian’s experiment, mangiferin improved inflammation in the DHEA-induced PCOS rat model by reducing the pp65/p65 ratio and blocking NF-κB signaling and the expression of the inflammatory cytokines IL-6, IL-1β, and TNF-α. They also found that inhibition of inflammation improved IR symptoms in DHEA-induced PCOS rats (Qian et al., 2020). Their research showed that the utilization of mangiferin could be considered a potential therapeutic strategy for PCOS.

## 5 Alkaloids

Alkaloids are a class of compounds with various therapeutic effects that play a massive role in treating diseases (Peng et al., 2019; Bai et al., 2021; Rampogu et al., 2022). However, according to the literature, alkaloids rarely show potential in treating PCOS. This phenomenon indicates that research on alkaloids may provide an opportunity to treat PCOS (Table 3).

**TABLE 3:** Therapeutic effects of alkaloids on PCOS.

Compound	Detail	Cell lines/model	Dose	Application	Ref
Berberine	an increase in live birth rate and fewer side effects after *in vitro* fertilization (IVF) treatment, decreased total testosterone, FAI, fasting glucose, fasting insulin, and HOMA-IR, and increased the SHBG	Infertile women with PCOS	3 × 500 mg	*clinical study*	An et al. (2014)
	Regulated IRS-1 and mTOR signaling pathway	Serum samples and granulosa cells treated with IVF/Intracytoplasmic Sperm Injection-Embryo Transfer woman	100 μM	*In vitro*	Kuang et al. (2020)
	Regulated the PI3K/Akt and MAPK pathways to improve GLUT4	Letrozole-induced PCOS rats model	400, 200, or 100 mg/kg	*In vivo*	(Zhang et al., 2020b)
	Inhibited apoptosis and regulated the expression levels of TLR4, LYN, PI3K, Akt, NF-κB, TNF-α, IL-1, IL-6, and caspase-3	DHEA-induced PCOS rats model	150 mg/kg	*In vivo*	Shen et al. (2021a)
	Regulated the PI3K/Akt pathway to promote proliferation and inhibited apoptosis of ovarian granulosa cells	Letrozole-induced PCOS rats model	95, 190 mg/kg	*In vivo*	(Yu et al., 2021)
	Downregulated the levels of LPAR3 and αvβ3	Testosterone propionate-induced PCOS rats model	100, 200 mg/kg	*In vivo*	(Wang et al., 2021)
	Promoted the ubiquitination of SIRT-3 to activate the AMPK pathway	The KGN cell line	0, 12.5, 25, 50 μM	*In vitro*	Li et al. (2020)
	Regulated gut microbiotas and metabolites	DHEA-induced PCOS rats model	150 mg/kg	*In vivo*	Shen et al. (2021b)

PCOS patients usually present with IR, an excess of androgens, an inflammatory response, infertility, or miscarriage. Metformin and insulin sensitizers are usually used to relieve the above symptoms in the clinic. Berberine (BBR) is an isoquinoline alkaloid that is mainly isolated from TCMs, such as *Berberis vulgaris* L. and *Coptis chinensis* Franch. BBR is regarded as an insulin sensitizer, and An et al. discovered that BBR was similar to metformin in reducing fasting glucose, fasting insulin, HOMA-IR, and androgen and increasing SHBG. Moreover, women who received berberine were associated with an increase in the live birth rate and fewer side effects after *in vitro* fertilization (an assisted reproductive technology) treatment than metformin (An et al., 2014). In addition, BBR increases insulin sensitivity by regulating IRS-1 and the mTOR signaling pathway (Kuang et al., 2020). Likewise, BBR upregulated GLUT4 to alleviate IR through dual regulation of PI3K/Akt and mitogen-activated protein kinases (MAPKs) (Zhang et al., 2020b). Additionally, Shen et al. discovered that BBR might alleviate PCOS by inhibiting apoptosis and regulating the expression levels of toll-like receptor 4 (TLR4), tyrosine kinase (LYN), PI3K, Akt, NF-κB, TNF-α, IL-1, IL-6, and caspase-3, which contributed to the improvement of IR (Shen et al., 2021a). For infertility and miscarriage caused by PCOS, Yu et al. confirmed that BBR promoted proliferation and inhibited apoptosis of ovarian granulosa cells through the PI3K/Akt pathway, which is beneficial to improving the pregnancy rate (Yu et al., 2021). High levels of lysophosphatidic acid receptor 3 (LPAR3) and integrin αvβ3 led to reduced endometrial receptivity and subsequent miscarriage. Wang et al. revealed that intervention with BBR could downregulate the levels of LPAR3 and αvβ3 in PCOS rats (Wang et al., 2021). Furthermore, BBR alleviates ovarian glucose metabolism disorders in PCOS. Li et al. indicated that BBR activated the AMPK pathway by promoting the ubiquitination of SIRT-3 and resulted in ovarian cell glucose uptake to maintain ovarian glucose homeostasis (Li et al., 2020). Regarding the effects of the gut microbiota on PCOS, Shen et al. found that berberine improved the symptoms of PCOS by regulating the structure of the gut microbiota and its metabolites (including glutamine, unsaturated acids, and glucose), bringing about decreased *Firmicutes* and increased *Bacteroidetes* at the phylum level, as well as increasing glutamine and decreasing glucose and unsaturated acids (Shen et al., 2021b). Their study offered a new direction for berberine in the improvement of PCOS.

## 6 Summary and outlook

Although modern medical treatments, including drugs and surgery, play an important role in treating PCOS, their accompanying side effects further limit their clinical application. Phytochemicals are a vital source of drug discovery and show the potential to improve the symptoms of PCOS and even possess some preventive effects. In this review, we summarized the advancement of these bioactive phytochemicals isolated from TCM and medicinal plants in treating PCOS, including flavonoids, polyphenols, and alkaloids. These phytochemicals show therapeutic effects on PCOS supported by *in vivo* and *in vitro* studies, mainly depending on anti-inflammatory, antioxidation, improvement of hormone disorder and IR, and alleviation of hyperinsulinemia (Figure 2). For instance, the anti-inflammatory effect is achieved by inhibiting the combination of ox-LDL and TLR4 from avoiding NF-κB pathway activation, which then elevates the expression of proinflammatory cytokines such as IL-1β, IL-6, IL-12, and TNF-α; activating AMPK to inhibit the NF-κB pathway; and restraining UPR activation by decreasing the production of proinflammatory cytokines. The antioxidation effect is primarily triggered by the initiation of UCP1 to increase the interaction between adiponectin and AdipoR1, leading to the activation of AMPK-SIRT-1 and decreased ROS production. As a result, antioxidant enzymes (such as GST, CAT, and GSH) are enhanced, leading to the activation of Nrf2 to produce Hmox1 and NQO1, followed by the downregulation of ROS. For IR or hyperinsulinemia, these valuable phytochemicals exhibit a potential capacity to sensitize insulin receptors attributed to their antioxidant and anti-inflammatory features. These phytochemicals can activate AMPK to increase GLUT4 translocation to regulate GLUT4 and the PI3K/Akt signaling pathway to increase insulin sensitivity. Moreover, LH binding with LH-R is implicated in androgen production. Some phytochemicals, such as baicalein, apigenin, rutin, SPA, and RVT, can directly decrease LH levels, which decreases androgen release. Several individual reports found that inhibition of the expression of 3β-HSD and 17β-HSD reduced the levels of androstenedione and testosterone. Because of the B-ring structure of flavonoids, these flavonoids are identical to the substrates for the enzymes 3β-HSD and 17β-HSD. Therefore, the binding of 3β-HSD and 17β-HSD with endogenous steroids can be reduced, thus indirectly reducing the activities of 3β-HSD and 17β-HSD. It can also reduce the levels of androgens such as androstenedione and testosterone. The structure-activity relationship of flavonoids has been preliminarily studied according to the literature, and detailed information on the binding sites and forms of flavonoids in ER and AR is summarized in Table 4. Furthermore, inhibition of GnRH can decrease the level of testosterone; regulation of PI3K/Akt in decreasing the expression of CYP17A1 avoids the conversion of pregnenolone to DHEA; and downregulation of FSH inhibits the combination between FSH and the FSH receptor to reduce cAMP through the PKA pathway for downregulating aromatase, showing potential therapeutic effects on PCOS. In addition to the above advancements, some bioactive phytochemicals indicate that regulating the activities of GK and HK to increase ERα gene expression and inhibiting the IRE1α-XBP1 pathway to downregulate Ar, Cyp11α1 and Cyp19α1 gene expression are beneficial to the improvement of PCOS.

**TABLE 4:** Phytochemicals binding to androgen and estrogen receptors.

Compound	Binding receptor	Binding site	Binding type	Effect	References
Quercetin	AR^T877A^ (PDB ID 3rll)	Gln711 and Trp741	Cocrystallized with a bulky B-ring antiandrogen	Induce the nuclear translocation of AR, anti-androgenic activity	(D'Arrigo et al., 2021)
		Leu704, Asn705, Gln711, Gln738, Tyr739, Met743, and His874	H-bonds		
		Met743 and Met895	Hydrophobic interactions		
		Trp741	π-π contact		
Soy isoflavones	wild type AR (PDB ID 3l3x)	Asn705, Gln711, and Arg752	H-bonds	Induce the nuclear translocation of AR, anti-androgenic activity	
		Leu704, Met742, Met745, Phe764, Leu873, and Met895	Hydrophobic interactions		
	BF3 pocket of wild type AR (PDB ID 2ylp)	Glu837, Glu829, and Asn833	H-bond		
		Phe673	π-π contact		
		Lys720, Gln733, Gln738	Interacting		
		Val716 and Met734	Hydrophobic interactions		
Naringenin	ERα (PDB ID 3uud)	Glu353, Arg394, Gly521, and Met421 or, alternatively, His524	H-bonds	Anti-androgenic activity	
	ERRβ (PDB ID 6lit)	Glu250, Arg291, Tyr301, Ala406	polar contacts		
		Phe410, Leu320, Leu243, Met281, Leu284, Ile288	Hydrophobic interactions		
		Phe410	π-π contact		
	ERRγ(PDB ID 2e2r)	Glu275, Arg316, His203, and Asn346	H-bonds		
Apigenin	ERRβ (PDB ID 6lit)	Glu250, Arg291, Tyr301, Ala406	polar contacts	Anti-androgenic activity	
		Phe410, Leu320, Leu243, Met281, Leu284, Ile288	Hydrophobic interactions		
		Phe410	π-π contact		
Luteolin	AR (PDB ID 2ylp)	Asn727	Contact	Anti-androgenic activity	
	ERRγ(PDB ID 2e2r)	His409	Contact		
Resveratrol	ERα (PDB ID 3uud)	Glu353, Arg394, Gly521, and Met421 or, alternatively, His524	H-bonds	Anti-androgenic activity	

In brief, flavonoids reduce androgen levels and improve hyperandrogenism mainly by inhibiting the activities of 3β-HSD, 17β-HSD, and aromatase, thereby inhibiting androgen conversion. For example, quercetin decreases the activities of 3β-HSD and 17β-HSD to reduce testosterone and estradiol levels. Regretfully, only a limited number of clinical studies have been designed to evaluate the efficacy of flavonoids in treating PCOS. Due to their phenolic groups, polyphenols have redox properties, which are responsible for antioxidant effects in treating PCOS. In addition, polyphenols also reduce the release of inflammatory factors by downregulating NF-κB, thereby achieving anti-inflammatory effects. For example, resveratrol, which affects the SIRT-1 pathway by mediating the deacetylation of p66Shc, reduces ROS to achieve antioxidant effects. The anti-inflammatory effect of resveratrol is to reduce the release of inflammatory factors by affecting the UPR process. However, the poor solubility and bioavailability of polyphenolic compounds, such as curcumin, hamper their clinical application. For alkaloids, berberine increases insulin sensitivity and alleviates IR by regulating IRS-1 and activating the PI3K/Akt pathway. Nevertheless, other valuable alkaloids have received less attention in treating PCOS. Of note, according to the literature, we detected those flavonoids and polyphenols accounted for most PCOS therapies, compared to a minor proportion of alkaloids. Continuous investment and attention to flavonoids and polyphenols will benefit the discovery of new drugs for PCOS.

There is a close relationship between hormonal alterations and bone metabolism in PCOS. For example, an increase in androgen leads to an increase in inflammatory factors and impairs bone formation. Estrogen is mainly used to maintain the development of female bones, while the decrease in estrogen in PCOS will cause some damage to bones. Other hormonal alterations, such as changes in LH, FSH, and insulin levels, also affect bone formation and development (Krishnan and Muthusami, 2017). Interestingly, some of the phytochemicals can directly or indirectly affect bone metabolism. For example, quercetin and curcumin can specifically mediate bone metabolism and osteoclast-related pathologies (Bian et al., 2021; Inchingolo et al., 2022); soy isoflavones can directly participate in bone metabolism and maintain the development of bones (Uehara, 2013; Pawlowski et al., 2015); resveratrol can stimulate the proliferation and differentiation of OB cells or inhibit the osteoclastic resorption (Vidoni et al., 2019; Inchingolo et al., 2022); and berberine can not only directly participate in bone metabolism, but also reduce the production of adipocytes (Jia et al., 2019). Notably, there is an inverse relationship between adipocytes and osteoblasts in bone marrow, which can indirectly affect bone metabolism by reducing the production of bone marrow adipocytes (Rayalam et al., 2011). As a result, these phytochemicals can potentially treat PCOS by affecting hormone and bone metabolism (Table 5). This conjecture needs to be validated by abundant experiments.

**TABLE 5:** Phytochemicals and bone metabolism.

Compound	Impact of bone	Cell lines/model	Dose	Application	Ref
Quercetin	Promoted bone marrow mesenchymal stem cell proliferation and osteogenic differentiation	Human bone marrow mesenchymal stem cells (BMSCs)	1, 5, and 10 μM	*In vitro*	(Bian et al., 2021)
Soy isoflavones	Inhibited bone resorption and improved bone calcium retention	Menopausal women	52.85, 113.52 mg	*Clinical study*	(Pawlowski et al., 2015)
Resveratrol	Stimulated OB cells proliferation and differentiation or inhibited osteoclastic resorption	Human Gingival Mesenchymal Stem Cells (HGMSCs)	1–100 μM	*In vitro*	(Vidoni et al., 2019)
Curcumin	Inhibited effects on the process of osteoporosis	100 patients with Spinal cord injury	110 mg/kg	*Clinical study*	(Hatefi et al., 2018)
	Pomoted osteogenic differentiation and inhibited adipogenic differentiation of hBM-MSCs	Human bone marrow-derived MSCs (hBM-MSCs)	0.05, 0.5 and 5 μM	*In vitro*	(Yang et al., 2021)
Berberine	Reduced alveolar bone loss and improved bone metabolism	Ovariectomized -periodontitis rats	120 mg/kg	*In vivo*	(Jia et al., 2019)

In addition to these encouraging outcomes, some problems need to be solved. Based on our review, these phytochemicals displayed therapeutic effects in alleviating the symptoms of PCOS. However, most of them are still in the preclinical research stage, except for quercetin, soy isoflavones, berberine, resveratrol, and cinnamon. The following reasons may explain this phenomenon. First, unsatisfactory bioavailability hinders their development in clinical application. A few compounds, such as curcumin, need to overcome poor bioavailability before entering clinical studies (Patel et al., 2020; Raja et al., 2021). In addition to chemical modification, advanced drug delivery systems such as nanoparticles (Luther et al., 2020; Nile et al., 2020) and antibody-conjugated drugs (Drago et al., 2021) can effectively solve the challenges of low bioavailability and precise drug delivery to tissues and cells, avoiding severe side effects. However, until now, only limited attention has been given to these valuable compounds. Conduction of the research above could accelerate the transformation of these powerful and valuable phytochemicals into clinical applications. Second, we still lack in-depth research on the pathogenesis of PCOS regardless of the known symptoms and possible mechanisms. Interestingly, a few studies have shown that gut microbiota and its metabolites significantly impact the evolution of PCOS, such as soy isoflavones, naringenin, and berberine, which can alleviate the symptoms by restoring the gut microbiota of PCOS models to a normal state. Further exploring the relationship between gut microbiota (Aron-Wisnewsky and Clement, 2016; Schuijt et al., 2016; Zuo and Ng, 2018) and PCOS may become a new research direction. Third, we found that many genes related to ovarian function were responsible for the evolution of PCOS, such as ERα, Ar, Cyp11α1, and Cyp19α1 genes. Nevertheless, current research on these valuable phytochemicals mainly focuses on the molecular level, except for quercetin, baicalin, apigenin, rutin, anthocyanins, and resveratrol. Hence, exploring the effects of other phytochemicals on PCOS at the genetic level might offer a different solution (Ruth et al., 2020; Mimouni et al., 2021) to treating PCOS in the future. Fourth, these molecules may play a different role in regulating PCOS than traditional small-molecule drugs. In recent years, the rise of targeted protein degradation (TPD) (Schapira et al., 2019; Dale et al., 2021) technology has revolutionized small molecule drugs, especially proteolysis-targeting chimeras (PROTACs) (Burslem and Crews, 2020; Bekes et al., 2022) and molecular glues (Mayor-Ruiz et al., 2020; Dale et al., 2021). These breakthrough technologies inspired us to determine whether these bioactive molecules play similar roles in alleviating or treating PCOS. Of course, sufficient experiments need to be conducted to test this hypothesis. In brief, this review aims to provide detailed mechanisms of these bioactive phytochemicals and hopefully practical and reliable insight into clinical applications concerning PCOS.
